# Sex‐Dependent Differences in Colonoscopy Sedation: Protocol for a Scoping Review

**DOI:** 10.1111/aas.70051

**Published:** 2025-08-26

**Authors:** Niels Brink Mylin, Veronica Ott, Ann Merete Møller, Birgitte Brandstrup

**Affiliations:** ^1^ Department of Clinical Medicine, Faculty of Health Science University of Copenhagen Copenhagen Denmark; ^2^ Department of Surgery Holbæk Hospital, Part of Copenhagen University Hospitals Holbæk Denmark; ^3^ Department of Anesthesia Herlev University Hospital Copenhagen Denmark

**Keywords:** colonoscopy, CQIs (caecal intubation rate etc.), sedation, sex/gender

## Abstract

**Aim:**

This scoping review aims to map evidence on sex differences in colonoscopy sedation use and their impact on colonoscopy quality indicators (CQIs).

**Methods:**

The review will adhere to the Preferred Reporting Items for Systematic Reviews and Meta‐Analyses extension for Scoping Reviews (PRISMA‐ScR).

The search will be conducted using MEDLINE, EMBASE, and the Cochrane Central Register of Controlled Trials. Only original studies investigating sex‐based differences in the use of sedation during colonoscopy and their influence on CQIs will be included. Data will be charted based on trial characteristics, demographic data, population size, sedation type, investigated gender‐dependent outcomes, and presented level of evidence.

**Results:**

A structured overview will be presented as a narrative summary with supplementary statistics.

## 1 Introduction

### 1.1 Rationale

Cancer surpassed cardiovascular disease as the leading cause of death globally in 2022 [1]. Among cancers, colorectal cancer (CRC) accounted for 1.9 million new cases worldwide that year, according to the World Health Organization (WHO). Early detection and adherence to treatment guidelines can significantly reduce CRC mortality [1]. Colonoscopy, as the gold standard for examining the colon and rectum, plays a crucial role in this effort, reducing CRC incidence by 50% and mortality by 40% through effective detection of polyps and early detection of cancers [2, 3].

Experts have established key quality indicators (CQIs) for colonoscopy to ensure optimal examination quality and patient outcomes. These indicators span three phases: preprocedural preparation, the examination itself, and postprocedural care, along with outcome measurements. The indicators primarily used for assessing procedural quality are the adenoma detection rate (ADR) and polyp detection rate (PDR). Other key indicators are the achievement of caecal intubation (the caecal intubation rate; CIR), adequate withdrawal time (WT), bowel preparation quality (BW), and complication rate (CR) [4, 5, 6]. Implementing CQIs in clinical practice and using them as a quality control measures for colonoscopies is strongly recommended [6]. A number of studies on colonoscopies could not show a difference in CIR or the time taken to reach the caecum between patients under moderate sedation and unsedated patients [7, 8]. Other studies have found increased CIR or ADR associated with sedation during colonoscopy [9, 10, 11, 12].

While sedation during colonoscopy significantly improves patient comfort and satisfaction, it also has notable drawbacks. Patients can experience impaired cognitive function as well as drowsiness after the procedure and are advised not to drive for up to 24 h. Most sedatives carry the risk of adverse events, with respiratory depression being the primary concern [13].

Studies consistently show sex differences in colonoscopy experiences and sedation preferences. A Polish cohort study of 22,750 patients who underwent screening colonoscopies (2014–2015) found that female patients reported more pain and discomfort than male patients, regardless of sedation status [7]. Similarly, a cohort study from Norway with 1198 cases (2011–2012) identified female sex as a significant risk factor for experiencing pain and discomfort during primarily unsedated colonoscopy [14].

A Saudi Arabian study, involving 403 colonoscopy patients (2011–2012), found that male patients were more inclined to choose unsedated colonoscopy than female patients [8]. These results are supported by other studies, which show that female patients both prefer and receive sedation during colonoscopy more frequently than male patients [9, 15].

A retrospective cohort study examined colonoscopy sedation practices in a vast American population: 1,385,436 colonoscopies performed from 2000 to 2013. Despite numerous studies investigating the influence of sedation on CQIs, the interaction between patient sex, sedation, and these indicators remains understudied.

### 1.2 Objective

This scoping review aims to investigate and outline the current understanding of how patients' sex influences the use of sedation during colonoscopy, as reflected in CQIs. The purpose of the review is to provide clinicians and researchers with an overview of the current state of evidence.

Research Questions:

1. Are there sex‐specific differences in sedation use and dosage during colonoscopy?
2. Does sex‐specific sedation affect colonoscopy quality indicators (CQIs)?
3. Which factors are known or hypothesized to affect potential sex differences in pain during colonoscopy?

## 2 Methods

### 2.1 Eligibility Criteria

This study will include peer‐reviewed primary research studies that investigate sex‐based differences in sedation use during colonoscopy.

Population: Patients (> 18 years) receiving colonoscopy, either elective or emergency, regardless of indication or diagnosis.

Concept: Sedation usage and sex‐dependent differences.

Context: Clinical studies across all areas of medicine and surgery.

The abstracts must include colonoscopy, sedation, sex differences, and CQIs (caecal intubation rate, adequate withdrawal time, quality of bowel preparation, complication rate) in different grammatical inflections. The abstracts must indicate quantitative data on the above‐mentioned subjects. All studies must report at least one sex‐specific difference.

Reviews, guidelines, commentaries, textbooks, editorials, letters, conference abstracts, and other non‐peer‐reviewed literature will be excluded, though they may be used to identify relevant primary research.

This study will include all relevant literature up to the publication date, regardless of the year of publication.

### 2.2 Information Sources

We will perform our search across the following databases: MEDLINE (indexing from 1947), EMBASE (indexing from 1946) and the Cochrane Central Register of Controlled Trials (no formal indexing cutoff). We will use Web of Science to identify grey literature and conduct forward and backward snowballing. This approach will help identify relevant studies not captured in our primary database searches.

The search was made in November 2024 and will be repeated prior to submission to ensure it captures the most current literature.

### 2.3 Search Strategy

The search strategy is designed with the assistance of a health sciences librarian.

The search string for EMBASE is provided in Table 1. Boolean operators (AND, OR) will be used to combine carefully selected Emtree terms into a search strategy. Since CQIs are not indexed as MESH or Emtree terms, we will limit the search to titles, abstracts, and keywords. Being standardized terms, they will not require additional synonyms.

**TABLE 1:** The search string for EMBASE.

Emtree terms	
Colonoscopy	Colonscop* OR coloscop*OR fiber colonoscop* OR Intesti* endoscop* OR Enteroscop* OR Ileocolonoscop* OR Colonoscop* surg* OR Colonoscop* surg* procedure*
Sedation OR conscious sedation OR deep sedation	premedication* OR an?esthesia pretreatment* OR an?esthesia premedication* OR prean?estheti* medic* OR prean?estheti* treatment* OR moderate sedati* OR sedative agent OR neurosedati* drug* OR sedati* OR sedati* drug* OR sedati* mix* OR soporific agent*
Sex/Gender	SEX* OR Gender* Male* OR m?n Female* OR wom?n
Colonoscopy Quality Indicators	adenoma detection rate* or polyp detection rate* or caecal intubation rate* or withdrawal time* or bowel preparation* or complication rate

*Note:* Boolean operators (AND, OR) will be used to combine the Emtree terms into a search strategy.

### 2.4 Data Management

Results from the primary search will be imported into Covidence [16] for screening and data extraction. A Microsoft Excel [17] spreadsheet will be used to record the information. Included studies will be catalogued and organized in Zotero [18].

### 2.5 Selection Process

Two independent reviewers will screen the titles and abstracts for eligibility, followed by a full‐text evaluation of the chosen articles in a similar fashion. Any disagreements will be resolved through discussion or, if needed, by involving a third reviewer.

### 2.6 Data Collection Process

The two reviewers will chart all included studies independently, followed by a comparison and discussion of their findings. Any disagreements that cannot be resolved between the two reviewers will be decided by a third independent reviewer. All authors of the scoping review will assess and discuss the results of the data extraction process.

A data extraction form will be created and pilot tested (Table 2).

**TABLE 2:** The data extracted into a form.

Category	Data
Study information	Author Year Study Design Setting Country
Patient information	Population Size Age Mean Age Median Sex (fem%)
Information on the colonoscopy	Indication Diagnosis CQI (Colonoscopy Quality Indicators); G(general) F(females) M(men): – ADR (Adenoma Detection Rate) – PDR (Polyp Detection Rate) – CIR (Caecel Intubation Rate) – WT (Adequate Withdrawal Time) – BW (Quality of Bowel Preparation) – CR (Complication Rate) Comments
Information on pain and outcome	Patient pain; G(general) F(females) M(men) Score Outcome Patient experience; G(general) F(females) M(men) Score Outcome
Information on sedation used	Sedation G(general) F(females) M(men) State (deep, conscious, general) Opioids Morphine Milligram Equivalents Benzodiazepines Approximately Equivalent oral dosage Other drugs
General pre‐ and postoperative information	Preoperative General information Postoperative General information
Key findings and Limitations	Key Findings Secondary Findings Limitations

### 2.7 Data Items

Data will be extracted and categorized into predefined sections, including, but not limited to: author, year, study design, setting, country, population (size, age, and sex) indication, diagnosis, sedation state, drugs, kind and dosage, gender differences, CQIs, and complications), pre‐, postprocedural information, key findings, secondary findings, and limitations (Table 2).

### 2.8 Outcomes and Prioritization

The primary outcome of this scoping review is to display the current literature regarding sex differences in sedation during colonoscopy, and their impact on CQIs.

We will present the findings in predefined subcategories:

- Reported sex differences in patient‐reported pain during colonoscopy.
- Sedation dosage differences between male and female colonoscopy patients.
- The impact of sex‐specific differences in sedation use on CQIs.

Secondarily, we aim to analyze whether anatomical or physiological factors are presented or theorized to affect colonoscopy pain.

Changes in the defined subcategories may be necessary to optimize the data charting process.

### 2.9 Risk of Bias in Individual Studies

We will identify potential sources of bias throughout the mapping process. These biases will be noted during the data charting process and reevaluated afterwards. This study is not intended to evaluate the quality of individual studies, as the primary goal of a scoping review is to map the evidence and identify strengths and gaps. To maintain this focus, we will provide a narrative evaluation of the biases and other limitations found in the reviewed literature.

### 2.10 Data Synthesis

The extracted data will be synthesized to emphasize similarities and differences. Analysis will be conducted to organize and categorize the identified sex differences.

## 3 Discussion

The proposed scoping review will examine and report the current knowledge on sex‐specific differences in pain and sedation during colonoscopy. The diverse material provided by web‐based search engines and the specialized nature of the topic pose a considerable challenge. A limitation of this scoping review is the entry date of the search terms. These dates may introduce bias through only reporting studies from 1946 (Medline) and 1974 (Embase) to the present. The specialized scope creates a risk of relevant sources being overlooked. To mitigate these challenges, we will use multiple search engines and conduct backward and forward snowballing.

## 4 Conclusion

Colonoscopy is considered the standard for examining the large intestine and rectum because it effectively identifies cancer, polyps, and various other lesions. The quality of the procedure can be dependent on proper sedation and reflected in CQIs, such as caecal intubation rate, adequate withdrawal time, quality of bowel preparation, and complication rate.

It has often been observed that women and men have different needs for and responses to this practice. The planned scoping review aims to identify sex‐specific sedation patterns and practices during colonoscopy and how they influence CQIs.

The objective is to inform researchers and clinicians about known and proven sex differences in colonoscopy‐related pain and approaches to address them. This could not only improve patients' experience but also optimize procedural quality.

## Author Contributions

All authors will meet the recommendations for authorship defined by The International Committee of Medical Journal Editors.

## Conflicts of Interest

The authors declare no conflicts of interest.

## Data Availability

No original data is generated in the making of this scoping review.
